# Founding Editorial — Atmospheric Systems and TheScientificWorld

**DOI:** 10.1100/tsw.2001.53

**Published:** 2001-06-15

**Authors:** Peter Brimblecombe, John G. Watson

## Abstract

There is a satisfying logic to the Greek choice of air, water, and earth as elements. Today we see this logic reflected in the way that that global science is subdivided into the categories of air, land, and water. Thus, the relevance of a science of global issues is not merely of academic interest. The tide of environmental concern, a vision of limits to growth, and a desire for sustainability have fostered an unprecedented interest in global sciences.

Atmospheric science has been the focus of particular attention, both scientific and societal. Urban pollution, regional haze, acid rain, the ozone hole, and the green house effect have become powerful verbal icons of our impact on the environment. Killer particles, El Niño, forest fires—all have attracted periods of media attention, often without an objective or informative scientific rationale.

The atmosphere has been a ready catalyst for changing public perception of the fragility of our environment. However, the reasons for this are related to the properties of the atmosphere that make it uniquely sensitive to human influence. It is the smallest of the major global reservoirs: a mere 5210 Eg (exagrams, Eg = $10^{18}$g). Massive, yet so much less so than the oceans or the crust that small amounts of materials released to such a small reservoir can have big effects. Furthermore, the rate of circulation and mixing is so much greater than for the oceans or the crust. The radioactive debris from Chernobyl was detected around the globe well within a half-life of a newspaper story.

To this we can add some interesting amplification effects. Phase changes in the atmosphere can lead to enormous concentration of materials. Acids in the atmosphere can be concentrated in rainwater perhaps a million-fold as they shift from the gas to the liquid phase. These high concentrations make catalysis, photochemistry, and reactions that are not first order more influential that they would be for dilute concentrations.

The atmosphere becomes a more complicated place as its physics and chemistry is better understood. This complexity is not a matter of mere scientific curiosity. Detailed knowledge of the radiative properties of greenhouse gases and aerosols is required if we are to understand the long-term impact human activities are likely to have on climate. The photochemistry of urban smogs shows that simple linear relationships between emitted pollutants and the resultant air pollution do not apply in all situations, involving us in more complex emissions reduction strategies of air quality management in our cities.

Increased scientific knowledge is required to maintain the quality of our environment. Although politicians often fail to understand the details of modern global sciences, few would publicly claim that they ignore its findings.

This balance between complexity and connectivity makes *TheScientificWorld* an excellent place for publishing materials about atmospheric science. *Atmospheric Systems* will be able to call a wide range of expertise from within its Board (listed below) and any number of other domains networked within *TheScientificWorld*. The editorial space aims to be a matrix with permeable boundaries between traditional scientific disciplines. This means it will be easy for readers to interact with the widest range of materials and scientists. More importantly, the connectivity will mean that the links will carry the ideas well into the area of applicability, such that we will be able to sense the political interface that has become such an important context for the atmospheric sciences.

Already within *TheScientificWorld* it is clear that air pollution scientists and the biomedical and life sciences communities will be able to interact on issues such as human health or biological or ecological impact. One of the first tasks of *Atmospheric Systems* is to create a bridge to these communities, to summarize the state of the science within main areas of research through authoritative review.

Review articles will be continually updated, and the information will be easy to link with materials lying within a wide range of databases. However, the types of information will be different as well. Traditionally, tabulated data within papers or those represented graphically have been in summary only. In *TheScientificWorld* the original data can be available on-line. This can be easily extended to provide on-line models, such that the reader can look at the way a published model responds to sensitive parameters. Output need no longer be static. In the on-line medium the traditional scientific illustration can become animated or molecules, structures, or asteroids can be rotated in three dimensions.

This provides a huge range of new possibilities within scientific publishing. It has the potential to be every bit as radical as the notion of the scientific journal created more than 300 years ago. Who knows where three centuries of development will take the principles established within *TheScientificWorld.*

Because of its great relevance to both contemporary science and society, *Atmospheric Systems* is an excellent platform for innovation. I do hope you will find your time spent with *TheScientificWorld* is helping to creating a new mode of scientific communication.

**Peter Brimblecombe**
**John G. Watson**
*Principal Editors*, *Atmospheric Systems*

May 2001

## Editorial Board for ATMOSPHERIC SYSTEMS A Domain of TheScientificWorld

### PRINCIPAL EDITORS

**Prof. Peter Brimblecombe**
School of Environmental Sciences, University of East Anglia, U.K.

**Prof. John G. Watson**
Division of Atmospheric Sciences, Desert Research Institute, U.S.A.

### ASSOCIATE EDITORS

**Dr. Harold Annegarn**
University of the Witwatersrand, Johannesburg, South Africa

**Prof. R. Balasubramanian**
Department of Chemical and Environmental Engineering, National University of Singapore, Singapore

**Dr. Sandro Fuzzi**
Instituto di Scienze dell'Atmosfera e dell'Oceano, Italy

**Prof. Rudolf B. Husar**
Washington University, U.S.A.

**Prof. N. Moussiopoulos**
Laboratory for Heat Transfer/Environmental Engineering, Aristotle University, Thessaloniki, Greece

### EDITORIAL BOARD MEMBERS

**Prof. Hajime Akimoto**
Institute for Global Change Research, Yokohama Campus, Japan

**Dr. Gregory P. Ayers**
CSIRO Atmospheric Research, Australia

**Dr. Zhipeng Bai**
Department of Environmental Science, Nankai University, P.R. China

**Dr. Jeff Brook**
Air Quality Process Research Division, Atmospheric Environment Service, Canada

**Dr. Judith C. Chow**
Desert Research Institute, U.S.A.

**Dr. Candis S. Claiborn**
Department of Civil & Environmental Engineering, Washington State University, U.S.A.

**Dr. Howard Hanson**
Los Alamos National Laboratory, U.S.A.

**Prof. Pham Duy Hien**
Ministry of Science, Technology and Environment, Vietnam

**Dr. Johann Engelbrecht**
Mineralogy Division, Mintek, South Africa

**Dr. Michael E. Jenkin**
AEA Technologies plc. National Environmental Technology Centre, U.K.

**Dr. William Malm**
National Park Services – Air Quality Division, Colorado State University, U.S.A.

**Prof. Sarah E. Metcalfe**
Department of Geography, University of Edinburgh, Scotland, U.K.

**Prof. Akira Mizohata**
Osaka Prefecture University, Japan

**Prof. Violeta A. Mugica**
Division de ciencias Basicas e Ingenieria, Universidad Autonoma Metropolitana, Mexico

**Prof. Rudolf Orthofer**
ARCS, Austria

**Prof. Ulrich Platt**
University of Heidelberg, Institute for Environmental Physics, Germany

**Dr. L. Willard Richards**
Sonoma Technology, Inc., U.S.A.

**Prof. Alexey Grigorievich Ryaboshapko**
Institute of Global Climate and Ecology, Russia

**Prof. Donald Wuebbles**
Department of Atmospheric Sciences, University of Illinois, U.S.A.

**Dr. Chung-Shin Yuan**
Institute of Environmental Engineering, National Sun Yat-Sen University, Taiwan, R.O.C.
